# *Listeria monocytogenes* infection in intestinal epithelial Caco-2 cells with exposure to progesterone and estradiol-17beta

**DOI:** 10.1371/journal.pone.0320631

**Published:** 2025-03-28

**Authors:** Anna Marie Hugon, Thaddeus G. Golos

**Affiliations:** 1 Wisconsin National Primate Research Center, University of Wisconsin-Madison, Madison, Wisconsin, United States of America; 2 Department of Pathology and Laboratory Medicine, University of Wisconsin-Madison, Madison, Wisconsin, United States of America; 3 Department of Comparative Biosciences, University of Wisconsin-Madison, Madison, Wisconsin, United States of America; 4 Department of Obstetrics and Gynecology, University of Wisconsin-Madison, Madison, Wisconsin, United States of America; Purdue University, UNITED STATES OF AMERICA

## Abstract

*Listeria monocytogenes* (Lm) is a food-borne pathogen associated with serious pregnancy complications, including miscarriage, stillbirth, preterm birth, neonatal sepsis, and meningitis. Although Lm infection within the gastrointestinal tract is well studied, little is known about the influence sex hormones may have on listeriosis. Estradiol-17beta and progesterone not only have receptors within the gastrointestinal tract but are significantly increased during pregnancy. The presence of these hormones may play a role in susceptibility to listeriosis during pregnancy. Caco-2 cell monolayers were grown on trans-well inserts in the presence of estradiol 17-beta (E2), progesterone (P4), both hormones, or no hormones (control). Cells were inoculated with Lm for 1 hour, before rinsing with gentamycin and transfer to fresh media. Trans-epithelial resistance was recorded hourly, and bacterial burden of the apical media, intracellular lysates, and basal media were assessed at 6 hours post inoculation. There were no significant differences in bacterial replication when directly exposed to sex steroids, and Caco-2 cell epithelial barrier function was not impacted during culture with Lm. Addition of progesterone significantly reduced intracellular bacterial burden compared to estradiol 17-beta only and no hormone controls. Interestingly, estradiol 17-beta only treatment was associated with significantly increased Lm within the basal compartment, compared to reduction in the intracellular and apical layers. These data indicate that the sex hormones P4 and E2 alone do not significantly impact intestinal epithelial barrier integrity during listeriosis, but that addition of P4 and E2, alone or in combination, was associated with reduced epithelial cell bacterial burden and apical release of Lm.

## Introduction

A ubiquitous environmental bacterium, *Listeria monocytogenes* (Lm) is primarily acquired via consumption of contaminated food that can cause gastrointestinal illness, meningitis, and sepsis [1]. The elderly, immunocompromised, neonates and pregnant people have increased susceptibility to listeriosis [2]. Gestational listeriosis is associated with serious pregnancy complications, including miscarriage, stillbirth, preterm birth, and neonatal sepsis and meningitis [3–6]. While Lm is known to cause significant fetal morbidity, it often goes unrecognized in the mother until infection at the maternal-fetal interface (MFI) causes adverse pregnancy outcomes (APOs) [7]. Furthermore, the determinants of increased susceptibility to infection with Lm during pregnancy remain undefined.

Within the GI tract, the gram-positive organism relies upon its dense peptidoglycan coating to survive the highly acidic environment of the stomach, until it reaches the intestinal villi where initial infection of the intestinal epithelium occurs [8,9]. Within the intestinal epithelium, Lm must overcome multiple barriers to infection including a host associated layer of mucus, the layers of epithelial enterocytes, immune cells, cytokines, metabolites, hormones, and other endogenous microorganisms, to achieve infection of the tissues lining the GI tract.

Intestinal epithelial tissues infected by Lm contain polarized cells that form a tight barrier to pathogen translocation. Tight junctions are essential for establishing a barrier to restrict the mixing of the two compartments [10]. Within the gut lumen, the intestinal epithelium forms a cellular layer between gastric contents and the circulatory systems of the body. Lm invades host cells through utilization of the bacterial surface proteins internalin A (InlA) and internalin B (InlB) [11]. Upon binding to epithelial cells, InlA and InlB activate signaling pathways of the host surface cell receptors and recruitment of cellular components needed for endocytosis. The signaling cascade leads to recruitment of actin and actin polymerization. During bacterial entry interactions with E-cadherin (Internalin A receptor) favor interaction of the E-cadherin cytoplasmic tail and the actin cytoskeleton, while entry via c-Met (Internalin B receptor) stimulates a signaling cascade to promote depolymerization of actin, which completes the process of bacterial internalization. It is worth noting that both of these receptors have been documented in colonic tissues [12,13]. Within the host cell, expression of ActA aids in polymerization of actin and generation of an actin tail to propel the bacterium through the cell cytoplasm and through membrane protrusions to enter neighboring cells [11]. Spreading within polarized epithelial cell layer is dependent upon internalin C (InlC) which promotes the formation of protrusions through inhibition of cellular Tuba and N-WASP which have been shown to modulate the structure apical junctions[14,15].

There are additional mechanisms that contribute to Lm crossing the intestinal epithelial barrier. Lm adhesion protein (lap) binding to Hsp60 initiates caveolin-1-mediated endocytosis of junctional proteins [16], activates cellular NF-KB and MLCK directing the redistribution of claudin-1, occludin, and E-cadherin in junctional complexes, which also fosters the accessibility of E-cadherin to InlA [17]. Junctional opening also can allow subsequent bacterial translocation (reviewed in ref. [18]).

By successfully invading the GI epithelial barrier through a cycle of replication and shedding into the gut lumen, Lm gains access to the lymphatic system through a process known as transcytosis [8,11,19]. Transcytosis is a form of cellular trafficking that Lm utilizes to move from one membrane to another, in this case from the apical layer to the basal layer of the GI epithelium, to ultimately enter the bloodstream [20]. Once in the bloodstream, Lm may circulate until it can access the intervillous space of the placenta, where molecules are transferred from mother to fetus including amino acids, fatty acids, glucose, and oxygen to underpin fetal development [21]. Lm in the intervillous space of the MFI is able to establish severe placental infection which ultimately causes acute inflammation, chorioamnionitis, and necrosis [22].

While the invasion and intracellular phases of Lm at the intestinal epithelial layer have been well described, the determinants of increased susceptibility to listeriosis during pregnancy remain unknown [23–28]. To evaluate these determinants, it is important to consider potential influences of the maternal gut environment on listeriosis.

During the course of a normal and healthy pregnancy, there are dramatic changes in the mother’s hormonal, metabolic, and immunological homeostasis. An important component of the pregnant state is elevation in the levels of the sex hormones E2 and P4 [29]. Circulating levels of P4 are elevated during the three trimesters of pregnancy, peaking during the third trimester, while the levels of E2 increase slowly before a rapid increase near the end of gestation [30]. These changes also are thought to alter immune responses during pregnancy [29,31–33]. During gestation, there is an overall decrease in pro-inflammatory cytokines and increase in counterregulatory cytokines [34]. These immune changes allow tolerance to potentially immunogenic paternal antigens in order to maintain a successful pregnancy. However, the loss in inflammatory signaling during specific stages of pregnancy creates a permissive state in which pathogen invasion does not trigger inflammation and a prompt robust immune response [29].

The modulation of inflammation plays an important role in not only the MFI, but also the GI tract. Researchers have demonstrated that sex steroids and theirs receptors serve an important role in the GI tract and contribute to the progression of a number of GI diseases, including inflammatory bowel disease (IBD), irritable bowel syndrome (IBS) and a variety of GI tract cancers [35–38]. IBD is an incompletely understood intestinal inflammatory disease, and is clinically characterized by a “leaky” GI tract, hypothesized to permit bacterial product translocation and inflammation [39]. Studies suggest that E2 may protect against acute colitis through the activation of ERβ [40,41]. One study demonstrated that treatment with E2 reduced inflammation in the colon in mice [42]. Research has also shown progesterone to inhibit IBD disease by improving gastrointestinal barrier function during pregnancy [43].

The presence of receptors for these hormones in intestinal epithelial cells has been long known [44–46], however their effects on epithelial barrier function during disease remains unclear. While studies have shown alterations to GI motility during pregnancy, evidence supports a mostly direct effect of P4 on intestinal smooth muscle, rather than progesterone-mediated pathways of gastric motility [36]. P4 has been documented to increase transepithelial electrical resistance in primary human colon tissues and Caco-2 cells through upregulating expression of the tight junction protein occludin [47]. In addition, E2 has been shown to decrease permeability through modulation of paracellular permeability and tight junctions in blood-brain barrier endothelia [48]. However, whether sex steroids affect the transcellular pathway in the intestinal epithelium remains undefined.

It is possible that circulating sex hormones impact the maternal gut microenvironment susceptibility to infection with Lm through incompletely defined mechanisms. We hypothesized that the presence of P4 alone would increase transepithelial electrical resistance (TEER) in the culture system, while E2 alone would decrease TEER. We also hypothesized that treatment with both P4 plus E2 would have decreased Lm within the intracellular and basal compartments. Receptors for both hormones have been documented in the human epithelial colorectal cancer (Caco-2) cell line making it an ideal model for examining the direct effects of P4 and E2 on Lm replication within the GI tract [35,44,46]. Our study aimed to build upon the existing knowledge by evaluating the impact of E2 and P4 during Lm infection of intestinal epithelial monolayers using a Caco-2 model.

## Materials and methods

### Cell culture

Human Caco-2 cells (HTB37; American Type Culture Collection) purchased from ATCC (Manassas, VA, USA) were maintained in Dulbecco’s Modified Eagle’s medium (DMEM, phenol red-free, Thermo Fisher Scientific, Waltham, MA, USA) supplemented with 20% charcoal-stripped FBS, 2 mM L-glutamine, 10 mM HEPES, 100 unit/mL penicillin and 100 µg/mL streptomycin (Thermo Fisher Scientific, Waltham, MA, USA) at 37°C under room air/5% CO_2_ in a humidified incubator. Cells were grown in 25 cm² culture flasks (Corning, Corning, NY, USA). The medium was changed every three days. The cells were harvested for passage or plating with 0.25% trypsin-EDTA (Thermo Fisher Scientific, Waltham, MA, USA).

To initiate infection experiments, the cells were harvested from confluent cell cultures and suspended in DMEM containing 10% fetal bovine serum and 1% non-essential amino acids. 24-well tissue culture plates containing 8 μm pore Polyethylene (PET) membrane inserts (catalogue number: 25-289, Corning, Corning City, NY, USA) were seeded with 3.5 ×  10^4^ cells per well and cultured to confluence with a final density of approximately 4 ×  10^5^ cells per well. Cells were then differentiated into a monolayer using Corning BioCoat Intestinal Epithelial Environment and protocol (catalog number: 355057, Corning, Corning City, NY, USA).

Hormone stocks of E2 or P4 were dissolved in ethanol to a stock concentration of 1 mg/mL, with further dilutions made in Modified Eagle’s medium (DMEM, phenol red-free, Thermo Fisher Scientific, Waltham, MA, USA) supplemented with 20% charcoal-stripped FBS, 2 mM L-glutamine, and 10 mM HEPES with no antibiotics. Wells were then treated with 2.50 ng/ml E2, 40 ng/ml P4, both, or no hormones as a control. Hormone concentrations were based upon circulating concentrations during pregnancy as reported in humans [49]. Since both the P4 and E2 were dissolved in absolute ethanol then diluted into media, an appropriate amount of ethanol was added to control wells. No impact of ethanol addition alone (<0.1%) was noted in preliminary studies. Cells were incubated in hormones for 24 hours prior to experimentation.

### Bacterial culture

**Fig 1:**
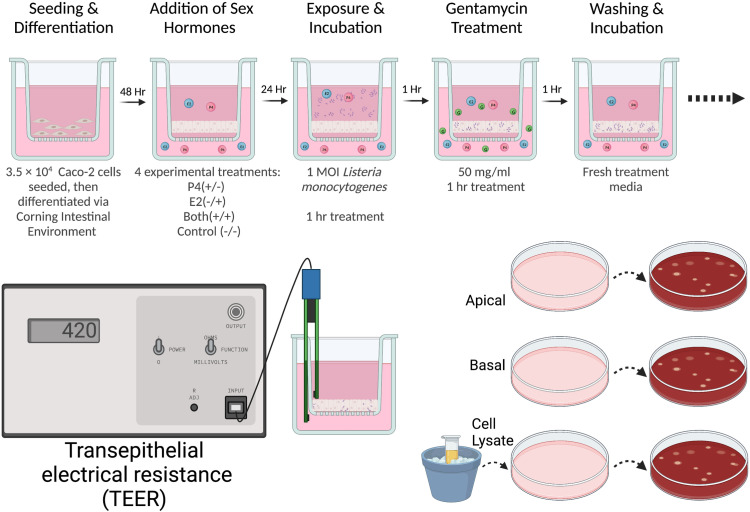
Image depicting the experimental timeline of each well. Each well features a transmembrane insert and media indicated by pink. Sex hormones are depicted by colored spheres, with E2 and P4 colored blue and pink, respectively. Gentamycin is represented by smaller green spheres, and Lm is denoted by purple rods. Following exposure, gentamycin, and washing, TEER was measured hourly for 6 hours then followed by plating of the apical media, basal media, and cell layer lysate on blood agar plates for quantification at 24 and 48 hours. Created with BioRender.com, under a CC BY license, original copyright 2023.

*L. monocytogenes* 2203S (wild type [WT]; serovar 4b [50]) was cultured overnight at 37◦C in Tryptic Soy Broth (TSB) (Becton Dickinson, Sparks, MD). Bacterial concentration was estimated using optical density measurements at a wavelength of 595 nm. Fresh bacterial cultures were washed and resuspended in Dulbecco’s modified Eagle medium (DMEM, phenol red-free, Thermo Fisher Scientific, Waltham, MA, USA) containing 10% fetal bovine serum (D10F) before addition to Caco-2 monolayers at a multiplicity of infection (MOI) of 1. The infectious dose was confirmed retrospectively by culturing tenfold serial dilutions of the inoculum in PBS on blood agar plates. Cells were incubated with Lm for 1 hour, then media were removed and replaced by D10F containing 50 μg/mL gentamicin (Thermo Fisher Scientific, Waltham, MA, USA) to kill extracellular bacterial cells (Fig 1). Gentamycin media were then removed, cells were washed, and transepithelial electrical resistance and infection studies were initiated.

### Barrier function

Barrier integrity was assessed using Trans-Epithelial Electrical Resistance (TEER) of polarized monolayers measured using the Millicell-Electrical Resistance System (ERS, Millipore, Billerica, MA, USA) and those with a minimum TEER of 200 Ω/cm^2^ (range, 200 to 600 Ω/cm^2^) were used for translocation experiments.

Pilot experiments were conducted to determine the appropriate length of time for monitoring bacterial translocation through Caco-2 monolayers, and hourly timepoints for 6 hours following exposure were chosen to capture the full progression of replication and translocation. Inserts with no cells as well as seeded inserts with no bacteria were used as negative controls for TEER. Each hormone treatment group was performed in duplicate wells and the experiment was replicated three times.

At 6 hours post exposure, the apical and basal media were separately collected, serially diluted, and plated on blood agar for the enumeration of Lm following 24 and 48 hours. The cells were resuspended in media and mechanically disrupted using the freeze-thaw method to release intracellular bacteria [51]. Lysates were serially diluted in PBS and plated on blood agar (Fig 1).

### Statistical analysis

All data analysis and graphs were prepared using Prism 9 software (Graph-Pad Software Inc., San Diego, CA, USA). Data from repeated experiments are presented as mean and standard error (SEM). Where appropriate, the Tukey multiple comparison test was used to identify statistically significant differences (P < 0.05).

## Results

### Bacterial replication

**Fig 2:**
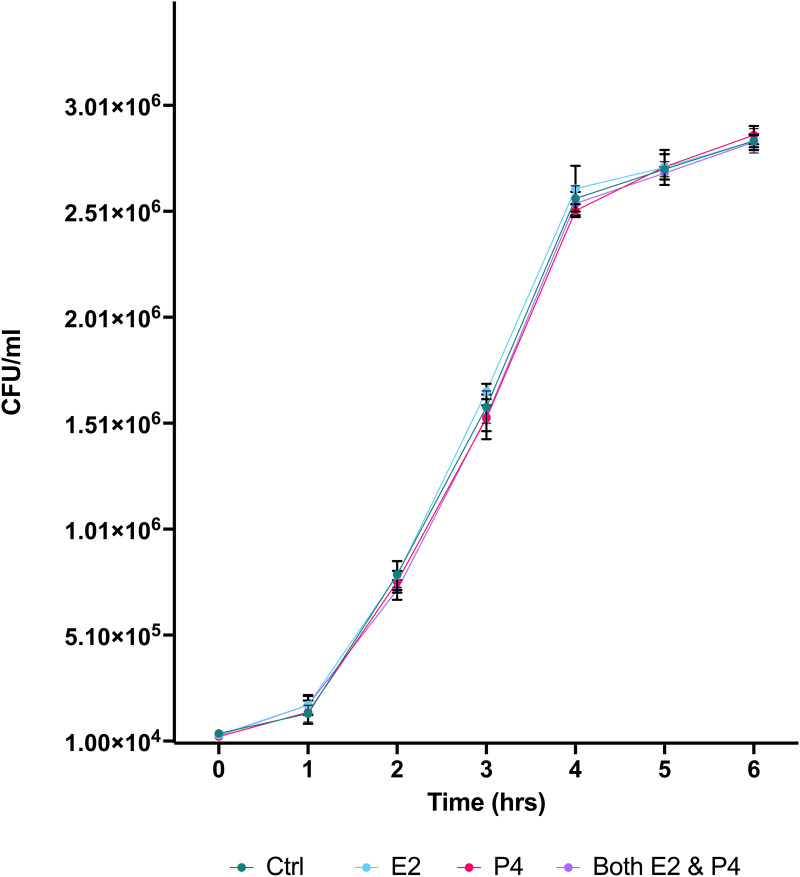
The growth of Lm (CFU/ml) during 6-hour incubation following exposure to E2, P4, both E2 and P4, or no hormone (control). The mean + /- standard error of the mean (SEM) is indicated.

First, we cultured Lm in TSB Media with 2.5 ng/ml E2, 40 ng/ml P4, both hormones, or no hormones (serving as control) to determine the impact of each treatment on survival and replication of Lm. 10^4^ CFU Lm were added followed by incubation at 37 °C. Bacterial levels (CFU/ml) of Lm was assessed using OD595 hourly for 6 hours following addition of Lm to hormone treated media. ANOVA with Turkey’s multiple comparison test found no significant differences in bacterial levels between hormonal treatment groups during 6h of culture (Fig 2).

### Barrier function

**Fig 3:**
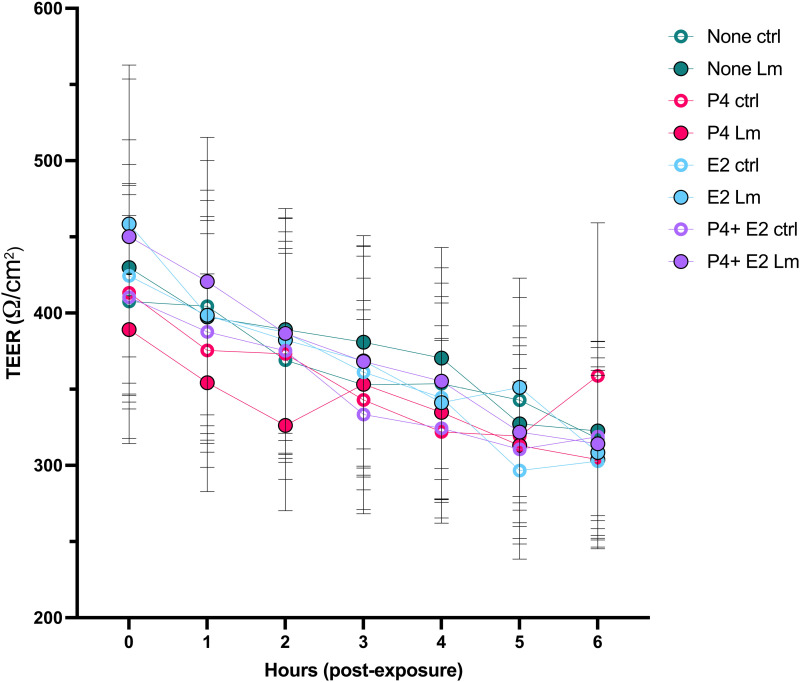
The graph depicts the average TEER values of cells grown on 8 μm pore inserts for 6 hours post-exposure to Lm. The treatment groups are color coded and Lm exposure is indicated by a filled symbol. The mean + /- SEM is presented.

We then examined the impact of hormone treatment on intracellular replication and barrier function with transcytosis of Lm by culturing cells on Transwell filter inserts on which Caco-2 cells were cultured to confluence and treated with media containing E2, P4, both, or neither hormone. Following 24 hours, 10^5^ CFU (1 MOI) of bacteria were added to the apical wells. The CFU of the inoculum was confirmed using culture-based methods. Trans-epithelial resistance (TEER) was recorded (Fig 3) and 2-way ANOVA and the Tukey multiple comparisons test of each treatment group compared with controls (no hormones) revealed no significant changes in barrier function with hormone treatment.

### Bacterial burden by compartment

**Fig 4:**
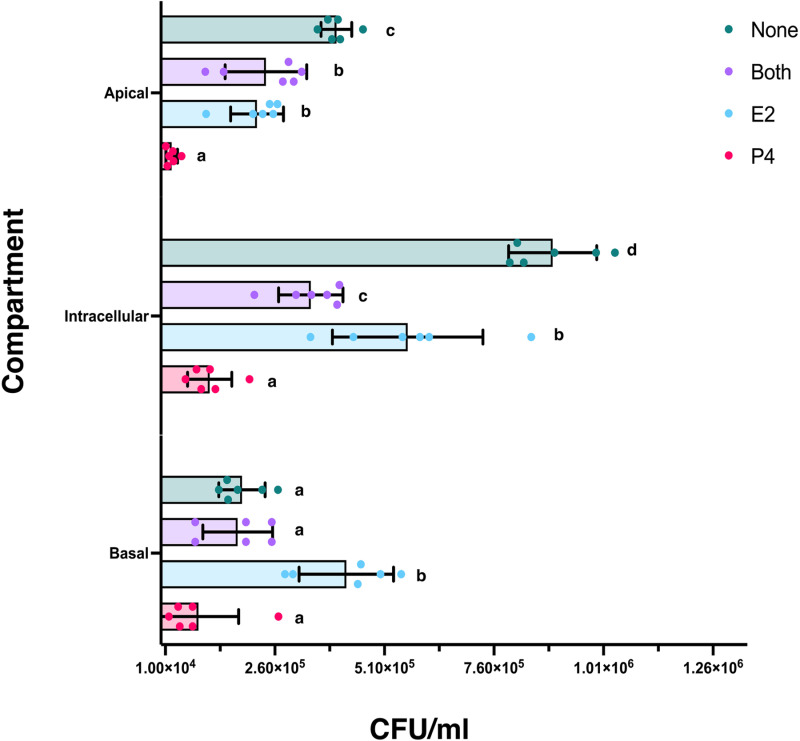
The scatterplot depicts the quantity of Lm (CFU/ml) in the apical layer, intracellular lysates, and basal layer. The treatment groups are color coded. The height of the bar indicates the mean of each treatment group, and the bars indicate standard error of the mean (SEM). Values that do not share superscripts are significantly different.

Following 6 hours incubation, the apical and basal compartment media were collected and plated to determine bacterial burden, the inserts were removed, and epithelial cells were lysed to determine intracellular bacterial burden (Fig 4). In the apical media, there was significantly less bacterial burden with P4 treatment only, compared to E2 only (p =  0.0014), E2 plus P4 (p =  0.0004), or no hormone controls (p =  < 0.0001). Both the E2 only (p =  0.0034) and both hormone treatment groups (p =  0.0112) also had significantly lower bacterial burden compared to no hormone controls.

The intracellular lysate had similar results, with significantly lower bacterial burden with P4 treatment only, compared to E2 only (p =  < 0.0001), E2 plus P4 (p =  0.0001), or no hormone controls (p =  < 0.0001). Within the lysates, the E2 only treatment group also had significantly more Lm compared to E2 plus P4 (p =  0.0002), but significantly less intracellular bacteria than the no hormone controls (p =  < 0.0001); cells treated with both hormones also displayed significantly lower bacterial burden than controls (p =  < 0.0001).

Within the basal media, the P4 only treatment group had significantly less bacteria compared to the E2 only treated group (p =  < 0.0001) and was lower than the E2 plus P4 or no hormones controls, although that difference did not achieve significance. Furthermore, the E2 only treated basal media had significantly increased bacterial burden when compared to either P4 only, E2 plus P4, or no hormone controls (p =  < 0.0001).

## Discussion

### Sex steroids and bacterial replication

In this study, we tested the ability of Lm to infect, and alter the barrier resistance of intestinal epithelial cell monolayers in the presence of P4 and E2 to elucidate the potential impact of pregnancy sex steroids on passage of Lm across the epithelium of the GI tract. While the intracellular phase of Lm infection has been extensively studied, little is known about the impact of sex steroids during infection of Lm within the GI tract. To rule out any effect of sex hormones directly on Lm replication, the impact of P4 and E2 on bacterial growth was first assessed in TBS culture. The data indicated that the replication of Lm is not directly affected by these hormones (Fig 2), confirming that sex steroids used in subsequent culture experiments do not directly impact Lm replication.

### Sex steroids and barrier function

To asses barrier function, we utilized a cell line derived from a colon adenocarcinoma (Caco-2), which differentiates into enterocyte-like cells under specific culture conditions and is a widely used model to assess intestinal epithelial permeability [52]. These cells develop into a polarized monolayer which form a border between the apical and basal compartments. Polarized epithelial cells are characterized by their ability to conduct endocytosis at either the apical or basal membranes [53]. The cell monolayer is organized similar to a honeycomb pattern, with junctions along all sides of the cells [52]. Tight junctions form the border at the basolateral cell surface domains in polarized epithelia, and support the maintenance of cell polarity by restricting intermixing of apical and basolateral transmembrane components [10]. Tight junctions include occludins and claudins, forming the zonula occludens [18]. These junctions modulate permeability between the intestinal epithelial cells, as well as intersect with signaling mechanisms that direct epithelial-cell polarization and the formation of apical and basal domains that are morphologically and functionally distinct [10]. Furthermore, the basal extracellular matrix constructed by epithelial cells in coordination with their junctions creates a barrier of separation between GI contents and circulatory networks of the body and is a barrier to bacterial dissemination into the bloodstream. This epithelial layer separates the contents of the intestinal lumen microenvironment of the GI tract including the GI contents, host-associated mucus, and microbial presence from the underlying circulatory system and lymphatic network of the intestinal villi. Each of these compartments has unique properties and represents various stages of Lm replication and release. The epithelial layer is where Lm replicates, and upon release of Lm from the cells the bacterium can freely reenter the lumen via the apical surface or escape the basolateral membrane to access the host circulation.

In our model, Lm readily infected Caco-2 cells and multiplied intracellularly but did not significantly decrease TEER of the Caco-2 monolayer as compared to uninfected cells. While there was no clear impact of sex steroids or Lm on transepithelial resistance, we found that within the apical and intracellular compartments, treatment with E2 or P4 independently led to a decrease in Lm burden. This effect of P4 was confirmed in the basal layer, with decreased Lm burden with P4 treatment, however treatment with E2 led to significantly increased Lm within the basal compartment, suggesting a more complex action of hormones on Lm passage across the epithelial barrier. Limitations of this culture system include that the epithelial environment in the gut *in vivo* is much more complex, including exposure to hormones over months, and that the adenocarcinoma cells may not accurately recapitulate the *in vivo* enterocytes.

Other studies examining the effects of P4 and E2 on Caco-2 barrier function found conflicting results. Salomon et. al examining Caco-2 cell brush-border membranes with P4 or E2 treatment found no effect on TEER of hormone treatment [54]. That study examined TEER function 5-, 10-, and 30-days following hormone treatment with P4 (310ng/mL) or E2 (270 ng/mL), with no differences in barrier function occurring at any of the time points. However, Zhou et al treated Caco-2 cells with 20 and 125 ng/mL of P4 for 24 h, and those authors found that TEER values were significantly increased following treatment with both P4 doses [47]. This was in part through an increase in occluding in Caco-2 tight junctions. Progesterone also impacts Caco-2 cells by inhibiting NF-KB activation by LPS. The endothelium of the blood-brain barrier is also responsive to progesterone, which increases occludin, claudin-5 and ZO-1 in those cells [48]. These data suggest that a longer treatment period is not required, but that increased concentrations of P4 may promote changes to barrier permeability. While we selected the dose of hormones in our experiments based on circulating levels in human pregnancy, future studies could examine the impact of other hormone doses prior to exposure to Lm.

In addition, Braniste *et al* [55] have reported that estradiol decreases gut permeability in rats. In Caco-2 cells, E2 or the estrogen receptor (ER) beta agonist diarylpropionitrile enhanced occludin and junctional adhesion molecule (JAM)-A expression without a change in ZO-1; this was blocked by the ER alpha antagonist ICI 182,780. Therefore, E2 reinforces the intestinal epithelial barrier through ERa and ERbeta regulation of paracellular spaces.

There is additional evidence that the intestinal epithelium is hormone-responsive. P4 suppresses cytokine-induced iNOS mRNA expression in Caco-2BBE and DLD-1 intestinal epithelial cells [56]. An ABC transporter molecule, P-glycoprotein (Pgp) functions to extrude drugs and toxins out of cells and limits intestinal uptake of drugs. Shulkin *et al* [57] showed that E2 treatment of Caco-2 cells increased Pgp synthesis and activity, whereas P4 increased synthesis, but decreased activity via nongenomic interactions with the molecule. Studies with the inhibitor ketoconazole showed that P4 acts through PXR, the pregnan-X receptor. Thus, there is ample evidence for direct action of sex steroids on enterocytes.

Another potential mechanism through which sex steroids could indirectly impact permeability is through modulation of cytokine signaling and inflammation. In the aforementioned study (47), the authors also examined serum from pregnant women during early gestation, late gestation, and post-partum stages and found that progesterone changes were associated with altered pro-inflammatory cytokine levels and showed that TNF-α, IL-6 and IL-1β were significantly reduced during the third trimester compared to postpartum [47]. These results indicate that the production of pro-inflammatory cytokines might be impacted through an indirect progesterone-mediated mechanism. A progesterone-mediated inhibition of pro-inflammatory cytokine action (e.g., [56]) could lead to decreased inflammation, diminished pathogen recognition, and immune cell activation, thus dampening the host response to infection. The impact of pro-inflammatory cytokines extends beyond immunologic activities and have been shown to affect epithelial barrier function [39,58–60], a key restraint on dissemination of Lm across the GI tract. Increased levels of P4 during pregnancy may prevent dissemination of Lm or reduce systemic inflammation through currently undefined mechanisms. We may not see an impact of hormones on TEER in our study as the Caco-2 cells are a limited model of the GI tract, i.e., only the intestinal epithelium. This does not account for changes to circulating cytokines as well as many other factors, including the complexity of the intestinal mucosa.

### Sex steroids and bacterial burden

Through quantification of Lm within the distinct basal, intracellular, and apical compartments of the Transwell culture system, we found a complex and significant impact of sex steroids on Lm replication and burden (Fig 4). In the apical media, addition of E2 or P4 independently led to a significantly decreased burden of Lm compared to controls. The combination of both hormones decreased Lm replication and release into the apical layer compared to controls, but the combination of E2 and P4 was not different from E2 alone, although significantly greater than P4 alone.

With intracellular lysates, either P4 or E2 treatment alone led to significantly reduced cellular Lm burden compared to controls. Treatment with both hormones also led to a significantly greater decrease in intracellular Lm compared to E2 only. This suggests that inhibition of Lm replication by P4 was additive to inhibition by E2, although the effect of P4 in the absence of E2 was also significantly greater than the effect of E2 plus P4.

The impact of sex steroids on Lm in the basal compartment was different from that seen in the apical or intracellular compartments. Lm burden in cells treated with E2 alone was statistically significantly higher than the other treatment groups. Lm burden in the P4 only group was similar to the apical levels, however the relatively lower control or E2 plus P4 groups resulted in no significant effect of P4 on Lm levels. This implies that there is differential release of Lm from the basal and the apical surfaces of the Caco-2 cells in this transwell chamber system. Collectively, across the apical, intracellular and basal compartments, these data confirm that P4 decreases Lm intracellular replication which is reflected by reduced release of Lm into the apical layer and reduced bacterial burden in the intracellular compartment.

In considering why the release of Lm into the basal compartment differs from the apical or intracellular compartments, one needs to consider Lm movement between and within epithelial cells. Motility of Lm within the epithelial layer is dependent on the host cell actin cytoskeleton and access to surface proteins such as E-cadherin and c-Met, which are normally on the basal side of intestinal cells [11]. Lm has been shown take advantage of apoptotic extrusion, which is a mechanism to remove dying or unwanted cells from an epithelium layer while preserving the barrier function [61]. Lm takes advantage of the temporarily exposed basal surface binding proteins to gain entry into the cell. Lm virulence factors such as InlC have been shown to further promote formation of cell protrusions, which aids in bacterial replication, comet tail assembly, and disrupts the structure of apical junctions in epithelial cells [15]. It is possible that E2 supports increased release of Lm into the basal compartment by indirectly altering and exposing tight junctions by increasing epithelial cell renewal and junction remodeling. E2 has been shown to have a protective effect on the gut epithelium, reducing inflammation through activation of Estrogen receptor-β (ERβ) [40–42]. ERβ expression in the GI tract has been reported to be higher in females compared to males [45]. Further, ERβ signaling has been shown to modulate epithelial barrier function [41,43,49]. One study demonstrated a reduction in ERβ mRNA expression and an increase in gut permeability prior to the onset of colitis in two animal models of spontaneous colitis [41]. The authors also used RT-PCR and electric cell-substrate impedance sensing of HT-29 and T84 colonic epithelial monolayers and found increased barrier resistance with E2 treatment. These data suggest that not only is more advanced modeling required, but that alterations to intestinal permeability are dependent on signaling pathways which are indirectly affected by the presence of sex steroids and their receptors.

We have shown that the pregnant macaque is more susceptible to listeriosis than the nonpregnant macaque [62], and we hypothesized that exposure of the Caco-2 cells to sex steroids would increase their susceptibility to infection and impair their barrier function. However, we did not find that P4 or E2 had this impact. There are several possible reasons for this discrepancy. It is possible that the acute short-term hormone exposure experiments feasible with the Caco-2 cell model do not fully mimic the long-term exposure of intestinal epithelial cells to sex steroids. Our previous study demonstrated susceptibility to listeriosis in early pregnancy, when the dam would have been exposed to elevated P4 and E2 for at least 6 weeks. The conditions for culture and maintenance of the Caco-2 cells preclude long-term exposure mimicking in vivo pregnancy.

Alternatively, while we tested the impact of sex steroids based on previous literature regarding this *in vitro* cell platform, it is possible that the other significant changes in the endocrine and immunological milieu of pregnancy affect the susceptibility of intestinal epithelial cells to *Listeria* infection. Future studies should also be expanded to evaluate other hormones which are associated with pregnancy, such as human chorionic gonadotropin (hCG), a well-studied placental hormone. HCG is one of the earliest secreted products of the conceptus and maintains the corpus luteum, which in turn supports progesterone secretion and pregnancy progression [63]. HCG has been shown to activate macrophages directly, and stimulate their innate immune functions [64]. It is possible that the changing levels of hCG throughout pregnancy, which peak during the first trimester of gestation, alter the maternal immune response to listeriosis. Some have proposed hCG as a potential anti-inflammatory therapy for sepsis, as it has been shown to display a wide range of significant anti-inflammatory effects [64–66]. However, the exact role (if any) of hCG during GI disease such as listeriosis remains undefined, and further research is needed into the impact of hCG or other pregnancy hormones on intestinal epithelial function.

## Conclusions

These data contribute to the beginning of our understanding of the determinants of susceptibility to listeriosis vis-a-vis the direct effect of sex steroids during Lm infection on the intestinal epithelial barrier. However, there are multiple elements to the barrier between the intestinal lumen and the host blood space. The basal extracellular matrix produced by epithelial cells and their lateral intercellular junctions creates a barrier of separation between GI contents and circulatory networks of the body. In our studies, presence of Lm within the basal layer represents successful intracellular replication and transcytosis across the intestinal tissue monolayer. We have demonstrated that Caco-2 cells are readily infected with *L. monocytogenes* in vitro and that there were no significant differences on the epithelial barrier function during listeriosis with exposure to sex steroids as measured by TEER. In addition, the data indicate that treatment with either E2 or P4 generally decreases Lm replication and release, although there may be differential steroid hormone regulation release of Lm into the apical and basal compartments was seen with E2 treatment alone.

These data suggest that increased susceptibility to listeriosis in pregnancy does not rely solely on the impact of circulating sex hormones on intestinal epithelial cell barrier function. Indeed, sex steroids actually appear to inhibit Lm replication in intestinal epithelial cells. It is well known that pregnancy is a risk factor for listeriosis, and that listeriosis in pregnancy is associated with a spectrum of adverse pregnancy outcomes. We have recently confirmed susceptibility to listeriosis in a pregnant macaque model, and that pregnancy also impacts susceptibility to gut dysbiosis in gestation [60]. It is possible that the maternal gut microenvironment may play a role in dispersion of Lm outside of the intestinal tract, with commensal microbes influencing Lm survival and invasion of epithelial tissue. Further studies are needed into the possibility that sex steroid-induced changes in the intestinal microbiome during pregnancy, or hormonal impact on other elements of the intestinal wall or immune system, may be involved in conferring increased susceptibility to listeriosis.

## Supporting information

S1 Table Statistical analysis of data underlying all figures. (XLSX)

## References

[1] Hof H. Listeria monocytogenes: a causative agent of gastroenteritis? Eur J Clin Microbiol Infect Dis. 2001;20(6):369–73. doi: 10.1007/pl00011277 11476434

[2] Pouillot R, Hoelzer K, Jackson KA, Henao OL, Silk BJ. Relative risk of listeriosis in Foodborne Diseases Active Surveillance Network (FoodNet) sites according to age, pregnancy, and ethnicity. Clin Infect Dis. 2012;54 Suppl 5:S405-10. doi: 10.1093/cid/cis269 22572661

[3] Silk BJ, Mahon BE, Griffin PM, Gould LH, Tauxe RV, Crim SM, et al. Vital signs: Listeria illnesses, deaths, and outbreaks -- United States, 2009-2011. MMWR: Morbidity and Mortality Weekly Report. 2013;62(10):448–52. doi: 10.15585/mmwr.mm6210a1 23739339 PMC4604984

[4] Charlier C, Disson O, Lecuit M. Maternal-neonatal listeriosis. Virulence. 2020;11(1):391–7. doi: 10.1080/21505594.2020.1759287 32363991 PMC7199740

[5] Lamont RF, Sobel J, Mazaki-Tovi S, Kusanovic JP, Vaisbuch E, Kim SK, et al. Listeriosis in human pregnancy: a systematic review. J Perinat Med. 2011;39(3):227–36. doi: 10.1515/jpm.2011.035 21517700 PMC3593057

[6] Sleator RD, Watson D, Hill C, Gahan CGM. The interaction between Listeria monocytogenes and the host gastrointestinal tract. Microbiology. 2009;155(Pt 8):2463–75. doi: 10.1099/mic.0.030205-0 19542009

[7] Pal M, Shuramo MY, Shiferawu F, Parmar BC. Listeriosis: An emerging food-borne disease of public health concern. Journal of Advances in Microbiology Research. 2022;3:29–33.

[8] Radoshevich L, Cossart P. Listeria monocytogenes: towards a complete picture of its physiology and pathogenesis. Nat Rev Microbiol. 2018;16(1):32–46. doi: 10.1038/nrmicro.2017.126 29176582

[9] Gahan CGM, Hill C. Gastrointestinal phase of Listeria monocytogenes infection. J Appl Microbiol. 2005;98(6):1345–53. doi: 10.1111/j.1365-2672.2005.02559.x 15916648

[10] Zihni C, Mills C, Matter K, Balda MS. Tight junctions: from simple barriers to multifunctional molecular gates. Nat Rev Mol Cell Biol. 2016;17(9):564–80. doi: 10.1038/nrm.2016.80 27353478

[11] Pizarro-Cerdá J, Kühbacher A, Cossart P. Entry of Listeria monocytogenes in mammalian epithelial cells: an updated view. Cold Spring Harb Perspect Med. 2012;2(11):a010009. doi: 10.1101/cshperspect.a010009 23125201 PMC3543101

[12] Kermorgant S, Cadiot G, Lewin MJ, Lehy T. Expression of hepatocyte growth factor and its receptor, C-Met in human digestive tissues and different gastric and colonic cancer cell lines. Gastroenterol Clin Biol. 1996;20(5):438–45. 8761141

[13] El-Bahrawy M, Poulsom R, Rowan AJ, Tomlinson IT, Alison MR. Characterization of the E-cadherin/catenin complex in colorectal carcinoma cell lines. Int J Exp Pathol. 2004;85(2):65–74. doi: 10.1111/j.0959-9673.2004.0371.x 15154912 PMC2517458

[14] Rajabian T, Gavicherla B, Heisig M, Müller-Altrock S, Goebel W, Gray-Owen SD, et al. The bacterial virulence factor InlC perturbs apical cell junctions and promotes cell-to-cell spread of Listeria. Nat Cell Biol. 2009;11(10):1212–8. doi: 10.1038/ncb1964 19767742 PMC2755649

[15] Otani T, Ichii T, Aono S, Takeichi M. Cdc42 GEF Tuba regulates the junctional configuration of simple epithelial cells. J Cell Biol. 2006;175(1):135–46. doi: 10.1083/jcb.200605012 17015620 PMC2064505

[16] Drolia R, Bryant DB, Tenguria S, Jules-Culver ZA, Thind J, Amelunke B, et al. Listeria adhesion protein orchestrates caveolae-mediated apical junctional remodeling of epithelial barrier for Listeria monocytogenes translocation. mBio. 2024;15(3):e0282123. doi: 10.1128/mbio.02821-23 38376160 PMC10936185

[17] Drolia R, Tenguria S, Durkes AC, Turner JR, Bhunia AK. Listeria Adhesion Protein Induces Intestinal Epithelial Barrier Dysfunction for Bacterial Translocation. Cell Host Microbe. 2018;23(4):470-484.e7. doi: 10.1016/j.chom.2018.03.004 29606495 PMC6750208

[18] Droila R, Bhunia AK. Crossing the Intestinal Barrier via Listeria Adhesion Protein and Internalin A. Trends in Microbiology 2019; 27: 408-425. 30661918 10.1016/j.tim.2018.12.007

[19] Becattini S, Pamer EG. Multifaceted Defense against Listeria monocytogenes in the Gastro-Intestinal Lumen. Pathogens. 2017;7(1):1. doi: 10.3390/pathogens7010001 29271903 PMC5874727

[20] Nikitas G, Deschamps C, Disson O, Niault T, Cossart P, Lecuit M. Transcytosis of Listeria monocytogenes across the intestinal barrier upon specific targeting of goblet cell accessible E-cadherin. J Exp Med. 2011;208(11):2263–77. doi: 10.1084/jem.20110560 21967767 PMC3201198

[21] Lamond NM, Freitag NE. Vertical Transmission of Listeria monocytogenes: Probing the Balance between Protection from Pathogens and Fetal Tolerance. Pathogens. 2018;7(2):52. doi: 10.3390/pathogens7020052 29799503 PMC6027155

[22] Bakardjiev AI, Theriot JA, Portnoy DA. Listeria monocytogenes traffics from maternal organs to the placenta and back. PLoS Pathog. 2006;2(6):e66. doi: 10.1371/journal.ppat.0020066 16846254 PMC1483233

[23] Corr SC, Gahan CGM, Hill C. Impact of selected Lactobacillus and Bifidobacterium species on Listeria monocytogenes infection and the mucosal immune response. FEMS Immunol Med Microbiol. 2007;50(3):380–8. doi: 10.1111/j.1574-695X.2007.00264.x 17537177

[24] Quereda JJ, Dussurget O, Nahori M-A, Ghozlane A, Volant S, Dillies M-A, et al. Bacteriocin from epidemic Listeria strains alters the host intestinal microbiota to favor infection. Proc Natl Acad Sci U S A. 2016;113(20):5706–11. doi: 10.1073/pnas.1523899113 27140611 PMC4878514

[25] Jaradat ZW, Bhunia AK. Adhesion, invasion, and translocation characteristics of Listeria monocytogenes serotypes in Caco-2 cell and mouse models. Appl Environ Microbiol. 2003;69(6):3640–5. doi: 10.1128/AEM.69.6.3640-3645.2003 12788773 PMC161501

[26] Lee S. Bacteriocins of Listeria monocytogenes and Their Potential as a Virulence Factor. Toxins (Basel). 2020;12(2):103. doi: 10.3390/toxins12020103 32033406 PMC7076858

[27] Mathipa MG, Thantsha MS, Bhunia AK. Lactobacillus casei expressing Internalins A and B reduces Listeria monocytogenes interaction with Caco‐2 cells in vitro. Microbial Biotechnology 2019; 12:715-729. 30989823 10.1111/1751-7915.13407 PMC6559204

[28] Ostling CE, Lindgren SE. Inhibition of enterobacteria and Listeria growth by lactic, acetic and formic acids. J Appl Bacteriol. 1993;75(1):18–24. doi: 10.1111/j.1365-2672.1993.tb03402.x 8365950

[29] Munoz-Suano A, Hamilton AB, Betz AG. Gimme shelter: the immune system during pregnancy. Immunol Rev. 2011;241(1):20–38. doi: 10.1111/j.1600-065X.2011.01002.x 21488887

[30] Smith R, Smith JI, Shen X, Engel PJ, Bowman ME, McGrath SA, et al. Patterns of plasma corticotropin-releasing hormone, progesterone, estradiol, and estriol change and the onset of human labor. J Clin Endocrinol Metab. 2009;94(6):2066–74. doi: 10.1210/jc.2008-2257 19258402

[31] Luk J, Seval Y, Ulukus M, Ulukus EC, Arici A, Kayisli UA. Regulation of monocyte chemotactic protein-1 expression in human endometrial endothelial cells by sex steroids: a potential mechanism for leukocyte recruitment in endometriosis. Reprod Sci. 2010;17(3):278–87. doi: 10.1177/1933719109352380 19933497

[32] Aydin Arici LMS, Seli E, Bahtiyar MO, Kim G. Regulation of monocyte chemotactic protein-1 expression in human endometrial stromal cells by estrogen and progesterone. Biol Reprod. 1999;61(1):85–90. doi: 10.1095/biolreprod61.1.85 10377035

[33] Fuhler GM. The immune system and microbiome in pregnancy. Best Pract Res Clin Gastroenterol. 2020;44–45:101671. doi: 10.1016/j.bpg.2020.101671 32359685

[34] Denney JM, Nelson EL, Wadhwa PD, Waters TP, Mathew L, Chung EK, et al. Longitudinal modulation of immune system cytokine profile during pregnancy. Cytokine. 2011;53(2):170–7. doi: 10.1016/j.cyto.2010.11.005 21123081 PMC4610033

[35] Chen C, Gong X, Yang X, Shang X, Du Q, Liao Q, et al. The roles of estrogen and estrogen receptors in gastrointestinal disease (Review). Oncology Letters. 2019. 10.3892/ol.2019.10983 PMC6865762 31788039

[36] Alqudah M, Al-Shboul O, Al-Dwairi A, Al-U´Dat D, Alqudah A. Progesterone inhibitory role on gastrointestinal motility. Physiological Research. 2022:193–8. 35344673 10.33549/physiolres.934824 PMC9150547

[37] Ueo H, Matsuoka H, Sugimachi K, Kuwano H, Mori M, Akiyoshi T. Inhibitory effects of estrogen on the growth of a human esophageal carcinoma cell line. Cancer Research. 1990;50:7212–5. 2224855

[38] Jacenik D, Cygankiewicz AI, Fichna J, Mokrowiecka A, Małecka-Panas E, Krajewska WM. Estrogen signaling deregulation related with local immune response modulation in irritable bowel syndrome. Mol Cell Endocrinol. 2018;471:89–96. doi: 10.1016/j.mce.2017.07.036 28774781

[39] Raffaella MG, Karel JvE, Bas O, Ellen CLW, Willem R, Stefania M, et al. Farnesoid X receptor activation inhibits inflammation and preserves the intestinal barrier in inflammatory bowel disease. Gut 2011; 60:463. 21242261 10.1136/gut.2010.212159

[40] Saleiro D, Murillo G, Benya RV, Bissonnette M, Hart J, Mehta RG. Estrogen receptor-β protects against colitis-associated neoplasia in mice. Int J Cancer. 2012;131(11):2553–61. doi: 10.1002/ijc.27578 22488198 PMC3404195

[41] Looijer-van Langen M, Hotte N, Dieleman LA, Albert E, Mulder C, Madsen KL. Estrogen receptor-β signaling modulates epithelial barrier function. Am J Physiol Gastrointest Liver Physiol. 2011;300(4):G621-6. doi: 10.1152/ajpgi.00274.2010 21252046

[42] Armstrong CM, Allred KF, Weeks BR, Chapkin RS, Allred CD. Estradiol Has Differential Effects on Acute Colonic Inflammation in the Presence and Absence of Estrogen Receptor β Expression. Dig Dis Sci. 2017;62(8):1977–84. doi: 10.1007/s10620-017-4631-x 28573506 PMC5751962

[43] van der Giessen J, van der Woude C, Peppelenbosch M, Fuhler G. P016 Pregnancy in IBD: direct effect of sex-hormones on epithelial barrier function. Journal of Crohn’s and Colitis. 2017;11(suppl_1):S87–8. doi: 10.1093/ecco-jcc/jjx002.142

[44] Thomas ML, Xu X, Norfleet AM, Watson CS. The presence of functional estrogen receptors in intestinal epithelial cells. Endocrinology. 1993;132(1):426–30. doi: 10.1210/endo.132.1.8419141 8419141

[45] Nilsson S, Gustafsson J-Å. Estrogen receptors: therapies targeted to receptor subtypes. Clin Pharmacol Ther. 2011;89(1):44–55. doi: 10.1038/clpt.2010.226 21124311

[46] Asavasupreechar T, Saito R, Miki Y, Edwards DP, Boonyaratanakornkit V, Sasano H. Systemic distribution of progesterone receptor subtypes in human tissues. The Journal of Steroid Biochemistry and Molecular Biology 2020; 199:105599. 31991170 10.1016/j.jsbmb.2020.105599 PMC9968951

[47] Zhou Z, Bian C, Luo Z, Guille C, Ogunrinde E, Wu J, et al. Progesterone decreases gut permeability through upregulating occludin expression in primary human gut tissues and Caco-2 cells. Sci Rep. 2019;9(1):8367. doi: 10.1038/s41598-019-44448-0 31182728 PMC6558054

[48] Si D, Li J, Liu J, Wang X, Wei Z, Tian Q, et al. Progesterone protects blood-brain barrier function and improves neurological outcome following traumatic brain injury in rats. Exp Ther Med. 2014;8:1010–4. 25120639 10.3892/etm.2014.1840 PMC4113529

[49] Schock H, Zeleniuch-Jacquotte A, Lundin E, Grankvist K, Lakso H-Å, Idahl A, et al. Hormone concentrations throughout uncomplicated pregnancies: a longitudinal study. BMC Pregnancy Childbirth. 2016;16(1):146. doi: 10.1186/s12884-016-0937-5 27377060 PMC4932669

[50] MacDonald PDM, Whitwam RE, Boggs JD, MacCormack JN, Anderson KL, Reardon JW, et al. Outbreak of listeriosis among Mexican immigrants as a result of consumption of illicitly produced Mexican-style cheese. Clin Infect Dis. 2005;40(5):677–82. doi: 10.1086/427803 15714412

[51] Didovyk A, Tonooka T, Tsimring L, Hasty J. Rapid and Scalable Preparation of Bacterial Lysates for Cell-Free Gene Expression. ACS Synth Biol. 2017;6(12):2198–208. doi: 10.1021/acssynbio.7b00253 28795570 PMC6038143

[52] Sun H, Chow ECY, Liu S, Du Y, Pang KS. The Caco-2 cell monolayer: usefulness and limitations. Expert Opin Drug Metab Toxicol. 2008;4(4):395–411. doi: 10.1517/17425255.4.4.395 18433344

[53] Apodaca G. Endocytic traffic in polarized epithelial cells: role of the actin and microtubule cytoskeleton. Traffic. 2001;2(3):149–59. doi: 10.1034/j.1600-0854.2001.020301.x 11260520

[54] Salomon R, Levy E, Levesque D, Szilagyi A, Seidman E. Caco-2 cell disaccharidase activities are unaffected by gestational hormones. Can J Physiol Pharmacol. 1996;74(10):1126–31. doi: 10.1139/cjpp-74-10-1126 9022832

[55] Braniste V, Leveque M, Buisson-Brenac C, Bueno L, Fioramonti J, Houdeau E. Oestradiol decreases colonic permeability through oestrogen receptor beta-mediated up-regulation of occludin and junctional adhesion molecule-A in epithelial cells. J Physiol. 2009;587(Pt 13):3317–28. doi: 10.1113/jphysiol.2009.169300 19433574 PMC2727039

[56] Salzman AL, Linn SC, Szabo C. Progesterone inhibits inducible nitric oxide synthase mRNA expression in human intestinal epithelial cells. Int J Mol Med. 2000;6(2):209–16. doi: 10.3892/ijmm.6.2.209 10891568

[57] Shchulkin AV, Chernykh IV, Popova NM, Slepnev AA, Yakusheva EN. Evaluation of female sex hormones influence on the protein-transporter p-glycoprotein functioning in vitro. Biomed Khim. 2020;66(6):444–9. doi: 10.18097/PBMC20206606444 33372901

[58] Cox AJ, West NP, Cripps AW. Obesity, inflammation, and the gut microbiota. Lancet Diabetes Endocrinol. 2015;3(3):207–15. doi: 10.1016/S2213-8587(14)70134-2 25066177

[59] Blaschitz C, Raffatellu M. Th17 cytokines and the gut mucosal barrier. J Clin Immunol. 2010;30(2):196–203. doi: 10.1007/s10875-010-9368-7 20127275 PMC2842875

[60] Elshaer D, Begun J. The role of barrier function, autophagy, and cytokines in maintaining intestinal homeostasis. Semin Cell Dev Biol. 2017;61:51–9. doi: 10.1016/j.semcdb.2016.08.018 27565684

[61] Gudipaty SA, Rosenblatt J. Epithelial cell extrusion: Pathways and pathologies. Semin Cell Dev Biol. 2017;67:132–40. doi: 10.1016/j.semcdb.2016.05.010 27212253 PMC5116298

[62] Hugon AM, Wolfe KB, Deblois CL, Simmons HA, Mejia A, Schotzo ML, et al. Listeria monocytogenes infection in pregnant macaques alters the maternal gut microbiome†. Biol Reprod. 2023;109(5):618–34. doi: 10.1093/biolre/ioad104 37665249 PMC10651077

[63] Kumar P, Magon N. Hormones in pregnancy. Niger Med J. 2012;53(4):179–83. doi: 10.4103/0300-1652.107549 23661874 PMC3640235

[64] Wan H, Coppens JMC, van Helden-Meeuwsen CG, Leenen PJM, van Rooijen N, Khan NA, et al. Chorionic gonadotropin alleviates thioglycollate-induced peritonitis by affecting macrophage function. J Leukoc Biol. 2009;86(2):361–70. doi: 10.1189/jlb.0208126 19414540

[65] Yoo SK, Mehdi SF, Pusapati S, Mathur N, Anipindi M, Lunenfeld B, et al. Human Chorionic Gonadotropin and Related Peptides: Candidate Anti-Inflammatory Therapy in Early Stages of Sepsis. Front Immunol. 2021;12:714177. doi: 10.3389/fimmu.2021.714177 34589085 PMC8475184

[66] Schumacher A, Heinze K, Witte J, Poloski E, Linzke N, Woidacki K, et al. Human chorionic gonadotropin as a central regulator of pregnancy immune tolerance. J Immunol. 2013;190(6):2650–8. doi: 10.4049/jimmunol.1202698 23396945

